# Pesticide seed dressings can affect the activity of various soil organisms and reduce decomposition of plant material

**DOI:** 10.1186/s12898-016-0092-x

**Published:** 2016-08-17

**Authors:** Johann G. Zaller, Nina König, Alexandra Tiefenbacher, Yoko Muraoka, Pascal Querner, Andreas Ratzenböck, Michael Bonkowski, Robert Koller

**Affiliations:** 1 Institute of Zoology, University of Natural Resources and Life Sciences Vienna (BOKU), Vienna, Austria; 2 Institute for Seed and Propagating Material, Phytosanitary Service and Apiculture, Austrian Agency for Health and Food Safety (AGES), Vienna, Austria; 3 Department of Terrestrial Ecology, Institute of Zoology, University of Cologne, Cologne, Germany; 4 Forschungszentrum Jülich, Institute of Bio- and Geosciences, IBG-2: Plant Sciences, Jülich, Germany

**Keywords:** Agricultural intensification, Agroecosystems, Belowground, Difenoconazole, Ecotoxicology, Fludioxonil, Imidacloprid, Pesticides, Prothioconazole, Soil ecology

## Abstract

**Background:**

Seed dressing with pesticides is widely used to protect crop seeds from pest insects and fungal diseases. While there is mounting evidence that especially neonicotinoid seed dressings detrimentally affect insect pollinators, surprisingly little is known on potential side effects on soil biota. We hypothesized that soil organisms would be particularly susceptible to pesticide seed dressings as they get in direct contact with these chemicals. Using microcosms with field soil we investigated, whether seeds treated either with neonicotinoid insecticides or fungicides influence the activity and interaction of earthworms, collembola, protozoa and microorganisms. The full-factorial design consisted of the factor Seed dressing (control vs. insecticide vs. fungicide), Earthworm (no earthworms vs. addition *Lumbricus terrestris* L.) and collembola (no collembola vs. addition *Sinella curviseta* Brook). We used commercially available wheat seed material (*Triticum aesticum* L. cf. Lukullus) at a recommended seeding density of 367 m^−2^.

**Results:**

Seed dressings (particularly fungicides) increased collembola surface activity, increased the number of protozoa and reduced plant decomposition rate but did not affect earthworm activity. Seed dressings had no influence on wheat growth. Earthworms interactively affected the influence of seed dressings on collembola activity, whereas collembola increased earthworm surface activity but reduced soil basal respiration. Earthworms also decreased wheat growth, reduced soil basal respiration and microbial biomass but increased soil water content and electrical conductivity.

**Conclusions:**

The reported non-target effects of seed dressings and their interactions with soil organisms are remarkable because they were observed after a one-time application of only 18 pesticide treated seeds per experimental pot. Because of the increasing use of seed dressing in agriculture and the fundamental role of soil organisms in agroecosystems these ecological interactions should receive more attention.

## Background

Seed dressing in agriculture involves the treatment of various crop seeds with fungicides and/or insecticides in order to combat soil borne fungal diseases and above- and belowground insects [1]. Neonicotinoid insecticides and fungicides used for seed dressing are increasingly applied for many agricultural crops for about 15 years [2, 3]. Recently, especially systemic neonicotinoid pesticides used for seed dressing have been shown to affect the fitness and mortality of a variety of non-target invertebrates [4, 5]. Especially their connection to increased bee mortality resulted in a moratorium on three neonicotinoids as seed dressing within the European Union [6]. While our knowledge on non-target effects of pesticide seed dressings on insect pollinators is mounting [5, 7], we still know very little on potential impacts on soil biota. This is surprising since the bulk of the active ingredients from seed dressings have been shown to enter the soil and thus directly impacting soil biota [2].

Of the highly diverse soil biota, earthworms are vitally important members especially in agricultural soils where they can constitute up to 80 % of total soil animal biomass [8]. They play critical roles in the development and maintenance of soil physical, chemical and biological properties [9]. Their activities improve soil structure by increasing porosity and aeration, facilitating the formation of aggregates and reducing compaction [10, 11]. Soil fertility is enhanced by earthworm casting activities [12] and the modification of microbial biomass and activity [13]. Collembola (springtails) are another very important part of soil fauna by driving plant litter decomposition processes [14, 15]. Other key components of the soil food web are heterotrophic protists (hereafter ‘protozoa’) that are involved in soil fertility and plant productivity as they remobilize nutrients formally locked in bacterial biomass [16, 17] and link energy fluxes towards higher trophic levels [18, 19].

Pesticides have been shown to affect earthworms from the physiological to community level, where insecticides and fungicides appear to be the most toxic pesticides [20, 21]. Recently, also broad-band herbicides have been demonstrated to impact earthworms and mycorrhizal fungi [22, 23]. In an extensive review on non-target effects of neonicotinoids several deleterious effects on soil organisms have been shown [24]. Neonicotinoids in seed dressings have been reported to decrease earthworm activity, burrowing and growth [25–28] and also affect terrestrial isopods [29] and soil microorganisms [30]. When a neonicotinoid was used as a lawn treatment to target neonate white grubs (Coleoptera: Scarabaeidae) an averaged 58 % reduction of non-target abundance of Hexapods, collembola, Thysanoptera and Coleoptera was seen [31, 32]. Several other studies also showed detrimental effects of neonicotinoids on collembola [33, 34]. Substantially less is known on potential side effects of fungicide seed dressings. However, as both earthworms and collembola feed on fungi living in the soil [35, 36] few studies indeed found that both collembola [37] and earthworms [38] can be affected by fungicide seed dressings. However, to our knowledge no study tested direct or indirect feedbacks on the impact of insecticide and/or fungicide seed dressings on Protozoa.

The aim of the present study was (i) to test the impact of insecticide and/or fungicide seed dressings on the activity or abundance of various soil biota ranging from microorganisms to macrofauna, (ii) to examine whether potential effects of seed dressings might be altered by the activity of soil meso and/or macrofauna (i.e. collembola or earthworms) and (iii) to quantify feedbacks of seed dressings on the functional capacity of soil biota to decompose plant litter. Because of their direct incorporation into the soil we hypothesized that pesticides in seed dressings will directly affect soil organisms of different functional and phylogenetic affiliations. Neonicotinoid insecticides will affect collembola because of their close phylogenetic relationship to insects and fungicides will indirectly affect earthworms and collembola as they both feed on soil fungi or by direct side effects. Including species interactions in potential non-target pesticide effects should provide a more realistic evaluation of the situation in agroecosystems [21–23, 39].

## Methods

### Study system

This experiment was conducted between 21 October and 16 December 2013 (58 days) in a greenhouse of the University of Natural Resources and Life Sciences (BOKU), Vienna, Austria. Experimental units, further called microcosms, consisted of polypropylene tubes (diameter 25 cm, height 60 cm) commonly used for sanitary tubing (type “PP-MEGA-Rohr 8”; Bauernfeind, Waizenkirchen, Austria). The bottoms of the tubes were closed with mosquito net and placed on saucers. Barriers of transparent plastic foil (20 cm high) were glued on the upper rim of each pot in order to prevent earthworms from escaping; these barriers were additionally smeared with soft soap on the upper edges.

Each microcosm was filled with 28.5 l of a substrate mixture made of 75 % (vol/vol) arable field soil and 25 % of commercial potting soil containing bark humus, wood fibres, compost of green waste, sand and mineral fertilizer (“green Pflanzerde”; BauMax, Klosterneuburg, Austria). Field soil was obtained from an arable field of the research farm of the University of Natural Resources and Life Sciences located in the village of Groß-Enzersdorf near Vienna, Austria. The two substrate types were thoroughly mixed using a concrete mixer. Characteristics of the substrate mixture: N_tot_ = 0.143 ± 0.05 g kg^−1^, P = 147.3 ± 13.8 mg kg^−1^, K = 289.5 ± 22.1 mg kg^−1^, C:N ratio 20.15, pH = 7.45 ± 0.02. Microcosms were randomly arranged on the floor of the greenhouse.

### Experimental factors

A full-factorial design with three factors was assigned to totally 60 microcosms; each factor combination was replicated five times.

Factor Seed dressing consisted of three levels of treated winter wheat seeds (*Triticum aestivum L. var. Lukullus*): No seed dressing, seed dressing with insecticides and fungicides (further called “insecticide seed dressing” because of the dominating insecticidal ingredients), seed dressing with fungicides only (further called “fungicide seed dressing”). Insecticide seed dressing consisted of the insecticide Gaucho^®^ 600 FS + Redigo^®^ (600 g/l imidacloprid + 100 g/l prothioconazole; Bayer CropScience; Monheim, Germany) combined with the fungicide CELEST^®^ Extra 050 FS (25 g/l difenoconazol, 25 g/l fludioxonil; Syngenta Agro, Vienna, Austria). Fungicide seed dressing consisted of EfA^®^UNIVERSAL (75 g/l fluoxastrobin, 10 g/l fluopyram, 7.5 g/l tebuconazole, 50 g/l prothioconazole; Bayer CropScience; Monheim, Germany). Control seeds had no dressing with pesticides. The seed material we used for this experiment was provided by the Austrian Agency for Health and Food Safety (AGES, Vienna, Austria) and is in this quality also available for farmers in Austria. We sowed 18 seeds per pot in 3 cm depth resulting in a density of 367 seeds m^−2^ which is within the recommended seeding density of 220–450 seeds m^−2^ for this variety (www.agrarvis.de/pflanzen). Variety *Lukullus* is regarded as quality wheat in Austria with excellent baking quality, high protein content particularly suitable for dry sites [40]. At the beginning, all microcosms were watered twice with 1.5 l of tap water to ensure maceration of seeds; afterwards all pots were regularly irrigated with the same amount of tap water depending on the temperature conditions in the greenhouse.

Factor earthworm consisted of two levels: addition of four adult individuals per microcosm (14.7 ± 2.1 g fresh mass) of the vertically burrowing species *Lumbricus terrestris* L. (+EW) or no earthworm addition (−EW). Adult specimens of *L. terrestris* were purchased from a bait shop (Anglertreff, Vienna, Austria) and acclimatized in field soil for 6 days in the climate chamber (15 °C) under complete darkness. Before introducing them to the microcosms, the earthworms were rinsed with tap water, dried with a hand towel and weighed. All earthworms buried themselves within a few minutes. One earthworm was lying dead on the soil surface 2 days after insertion and was immediately substituted by another one.

Factor collembola consisted of two levels and was established either by adding 100 *Collembola* of the species *Sinella curviseta* Brook, 1882 (*Entomobryidae;* treatment +C) to half of the microcosms immediately after seeding (21 October 2013) or by adding no collembola (treatment –C). Collembola were obtained from a commercial supplier (Megazoo, Vienna, Austria). To provide abundant food for earthworms and Collembola, 3.5 g microcosm^−1^ of chopped hay and 0.2 g microcosm^−1^ fish fodder (TetraMin^®^) was spread on the soil surface of each experimental unit over the cource of the experiment in order to keep the nutrient input similar between treatments.

The earthworm species used is native to Central European agroecosystems [41], the collembola species used is native to Europe, Southeast Asia (especially China) and north-western parts of the USA [37].

### Measurements

#### Earthworms

The activity of earthworms was assessed using the toothpick method [22]. Briefly, regular wooden toothpicks are vertically inserted into the soil (ca. 3 mm deep) before sunset, the next morning the inclined or fallen toothpicks were assessed. Vertically burrowing earthworms will come to the soil surface during night in order to forage for food and will thereby knock over toothpicks. We used 12 toothpicks per microcosm and conducted this assessment twice a week. Another method we used to assess earthworm activity was the counting of earthworm casts deposited on the soil surface. All surface casts were counted and collected twice a week. The casts were dried at 40 °C for 48 h and weighed.

#### Collembola

The activity of *Collembola* was determined using pitfall-traps [42]. Therefore, five uncovered 2 µl Eppendorf tubes (diameter 9.85 mm) were carefully inserted so deep that the upper rim of the tubes was at the level of the soil surface. Tubes were inserted around the centre of each microcosm using a consistent pattern among microcosms. Pitfall-traps were filled with conservation fluid consisting of 95 % ethylene glycol and a drop of odourless detergent. Sampling started 4 days after the addition of collembola on 25 October; after 4 days of exposure the pitfall-traps were replaced with new ones, which were exposed for another 4 days. Four sampling intervals each with a four-day exposure were made. Between 14 November and 16 December 2013 five samplings with six-day exposure interval were made. All specimens captured in the pitfall traps were stored in 95 % ethylene glycol at room temperature until they could be counted and assigned taxonomically.

In addition to the test organism two other *Collembola* species were found: two individuals of *Sminthurinus domestica* and one individual of *Entomobrya multifasciata*. Because these latter two species were so rare, they were excluded from further calculations. Daily *Collembola* activity was calculated by dividing the cumulated number of trapped *Collembola* by the number of days of pitfall trap exposure.

#### Soil moisture, electrical conductivity and temperature

These soil parameters were measured twice a week when assessing earthworm activity using time domain reflectrometry (TRIME^®^-PICO 64/32, Micromodultechnik GMBH, Ettlingen, Germany).

#### Wheat growth

Growth of winter wheat was assessed weekly on all 18 plants per microcosm by measuring the maximum leaf length from the soil surface using a ruler. Aboveground winter wheat biomass was destructively harvested on 16 December (58 days after seeding) by cutting all wheat plants at the soil surface. Wheat biomass was assessed after drying the plant material at 55 °C for 48 h.

#### Litter decomposition in soil

Litter decomposition in soil was determined using the Tea Bag Index [43]. Therefore, one commercially available pyramid shaped plastic tea bag of green tea (EAN: 87 22700 05552 5) and one tea bag of rooibos tea (EAN: 87 22700 18843 8) were buried at a depth of 8 cm in each microcosm (Lipton Tea, Washington St, USA). The mesh size of the tea bags of 0.25 mm allows microorganisms to enter, but meso and macrofauna are excluded [44]. Before the insertion into the microcosms individual tea bags were weighed, tea bags remained in the microcosms for 58 days. After the removal from the microcosms, the tea bags were cleaned from sticking soil particles and dried at 70 °C for 48 h. The bags were opened and the content was weighed. The calculation scheme determined the decomposition rate (k) and the stabilisation factor (S) considering the hydrolysable fraction 0.842 g g^−1^ for green tea and 0.552 g g^−1^ for rooibos tea [43]. Green tea and rooibos tea have different decomposition rates meaning that rooibos tea decomposes slower and still continues, when labile material in green tea has already been consumed. The stabilisation process begins during the decomposition of the labile fraction of organic material [45]. This method was also used to assess non-target effects of herbicides [23].

#### Soil microorganisms

Soil microbial biomass (C_mic_) was determined from a 3 g subsample of 20 g of fresh surface soil (0–3 cm) taken on three random locations per microcosm 54 days after seeding (12 December 2013). Soil was stored in polypropylene plastic bags, cooled and expressed-mailed to the University of Cologne, Germany, where the analyses on soil microbes were conducted. Microbial biomass was measured by substrate-induced respiration [46] using an automated respirometer based on electrolytic O_2_ micro compensation [47], as outlined in [48]. For basal respiration, the average O_2_ consumption rate of samples not amended with glucose was measured during 15–20 h after attachment of samples to the respirometer. Microbial specific respiration (qO_2_, µl O_2_ µg^−1^ C_mic_ h^−1^) was calculated as the quotient between basal respiration and microbial biomass.

For the quantification of Protozoa (Amoebae and Flagellates), soil samples were taken from the top 3 cm from three random locations per microcosm 54 days after seeding (12 December 2013). The soil was homogenized and stored at 5 °C until usage. Amoebae and Flagellates were counted using a modified most probable number method [49]. Briefly, 5 g fresh weight of soil was suspended in 20 ml sterile Neff’s modified amoebae saline (NMAS; [50]) and gently shaken for 20 min on a vertical shaker. Threefold dilution series with nutrient broth (Merck, Darmstadt, Germany) and NMAS at 1:9 v/v were prepared in 96-well microtiter plates (VWR, Darmstadt, Germany) with four replicates, each. The microtiter plates were incubated at 15 °C in darkness and the wells were inspected for presence of protozoa using an inverted microscope at 100× and 200× magnification (Nikon, Eclipse TE 2000-E, Tokyo, Japan) after 3, 6, 11, 19 and 26 days. Densities of protozoa were calculated according to [51].

#### Air temperature and relative humidity

Air temperature and relative humidity in the greenhouse was monitored using Tinytag dataloggers (Tinytag Plus 2, Gemini Data Loggers Ltd, Chichester, West Sussex, UK). Mean daily air temperature during the course of the experiment was 17.9 °C and at a mean relative humidity of 64.4 %.

### Statistical analyses

All statistical tests were carried out using R-software vers. R-3.0.2 for Windows (www.r-project.org). All data were tested for normal distribution by the Shapiro–Wilk test and homogeneity of variance by the Levene test. Three factorial analysis of variance (ANOVA) with the factors seed dressing, earthworms, collembola and their interactions was used to examine effects on wheat growth, wheat biomass, soil microbial parameters, litter decomposition, soil abiotic parameters. Two factorial ANOVAs with the factors seed dressing and collembola were used to test effects on total cumulated earthworm surface activity. Two factorial ANOVAs with the factors Seed dressing and Earthworms were used to test effects on total cumulated collembola surface activity. Posthoc Tukey comparisons were used to test effects of treatment factors at individual treatments. Differences were considered significant when P < 0.05 and marginally significant when 0.07 < P > 0.05. All values given in the text are means with the appropriate standard deviation (mean ± SD).

## Results

**Fig. 1:**
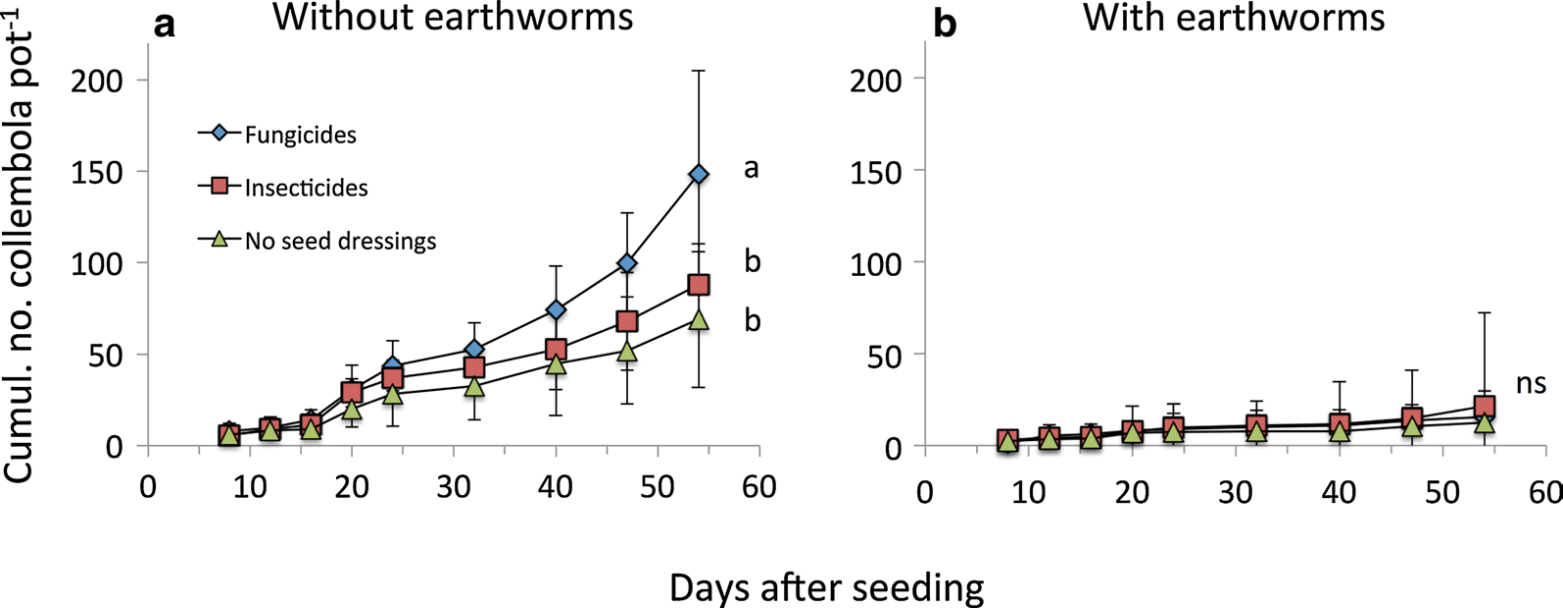
Collembola activity in response to pesticide seed dressings in microcosms without (**a**) and with earthworms (**b**). Mean ± SD, n = 5. *Different letters* denote significant differences between seed dressings, ns no significant difference

**Fig. 2:**
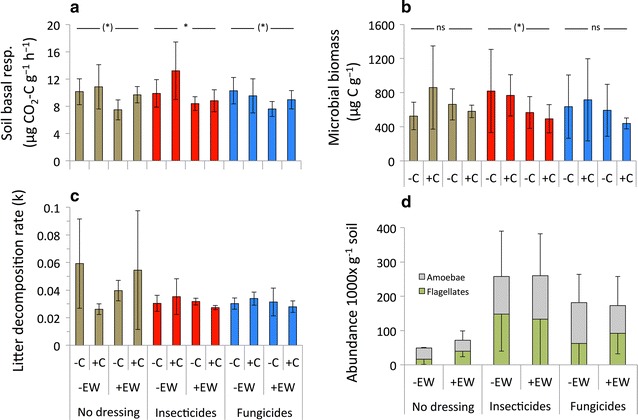
Soil basal respiration (**a**), microbial biomass (**b**), soil decomposition rate (**c**) and protozoa abundance (**d**) in response to pesticide seed dressings in microcosms without (−C) or with collembola (+C), without (−EW) or with earthworms (+EW). Mean ± SD, n = 5. *Horizontal lines* indicate mean comparisons between earthworm treatments when interactions were significant: * denotes significant difference, (*) marginally significant difference, *ns* no significant difference

Generally, we observed earthworm and collembolan activity throughout the course of the experiment. Seed dressing significantly increased the cumulated surface activity of collembola (Fig. 1; Table 1), decreased litter decomposition rates and marginally significantly increased the abundance of soil protozoa (Fig. 2; Table 1). Fungicide seed dressings increased cumulative collembola activity when earthworms were absent (Fig. 1a). Cumulative collembola activity was highest after fungicide seed dressing (148 ± 14 ind. pot^−1^), followed by insecticide seed dressing (88 ± 5 ind. pot^−1^;) and no seed dressing (69 ± 5 ind. pot^−1^, Fig. 1a). Collembola surface activity was unaffected by seed dressings when earthworms were present (i.e. significant seed dressing × earthworm interaction; Fig. 1b; Table 1). Daily collembola activity was significantly increased by fungicide seed dressings (averaged 4.12 ± 0.70 ind. pot^−1^ day^−1^) while insecticide seed dressing and non-treated seeds showed similar activities (2.44 ± 0.27 ind. pot^−1^ day^−1^ and 1.92 ± 0.46 ind. pot^−1^ day^−1^, respectively; data not shown). Litter decomposition rate was significantly reduced by both fungicide and insecticide seed dressings (on average 0.029 ± 0.006) and higher when no seed dressings were used (0.050 ± 0.026; Fig. 2c; Table 1). Both types of seed dressings marginally significantly increased protozoa densities (Fig. 2d; Table 1). All other soil or plant parameters measured remained unaffected by seed dressings (Table 1).

**Table 1:** ANOVA-results on the effects of seed dressings, earthworms and collembola on soil and plant parameter

Parameter	Seed dressing (SD)		Earthworms (EW)		Collembola (coll)		SD × EW		SD × coll		EW × coll	
	F	P	F	P	F	P	F	P	F	P	F	P
Earthworms												
Surface activity (toothpicks)	2.37	0.104	–	–	2.97	0.091	–	–	1.42	0.253	–	–
Surface activity (no. casts)	0.78	0.464	–	–	8.02	*0.007*	–	–	0.30	0.739	–	–
Surface activity (cast mass)	0.75	0.479	–	–	2.87	0.097	–	–	0.91	0.411	–	–
Collembola												
Surface activity (total no.)	5.04	*0.010*	62.56	<*0.001*	–	–	4.97	*0.011*	–	–	–	–
Surface activity (daily no.)	1.41	0.250	9.87	*0.003*	–	–	1.90	0.159	–	–	–	–
Protista												
Flagellates (abundance g^−1^ soil)	3.36	*0.053*	0.13	0.720	–	–	0.16	0.849	–	–	–	–
Amoebae (abundance g^−1^ soil)	1.54	0.237	0.03	0.855	–	–	0.17	0.842	–	–	–	–
Protozoa (abundance g^−1^ soil)	3.31	*0.055*	0.01	0.933	–	–	0.02	0.979	–	–	–	–
Soil microorganisms												
Basal respiration (µg CO_2_–C g^−1^ h^−1^)	1.01	0.372	14.794	<*0.001*	4.56	*0.038*	0.47	0.628	0.78	0.492	0.03	0.866
Microbial biomass C_mic_ (µg C g^−1^)	0.26	0.773	4.07	*0.049*	0.02	0.881	0.48	0.619	0.54	0.585	1.93	0.171
Metabolic quotient qCO_2_ (µg CO_2_–C g^−1^ h^−1^ C_mic_ h^−1^)	0.98	0.382	0.03	0.856	1.51	0.225	2.91	*0.064*	0.73	0.489	7.99	*0.007*
Litter decomposition												
Decomposition rate (k)	3.80	*0.043*	0.01	0.955	0.45	0.507	0.19	0.825	1.03	0.368	1.01	0.322
Stabilisation factor (S)	0.25	0.779	0.29	0.588	1.34	0.254	0.26	0.769	2.07	0.139	1.80	0.187
Soil abiotic parameters												
Water content (%)	1.98	0.149	20.83	<*0.001*	1.52	0.224	0.90	0.412	0.53	0.589	0.01	0.983
Temperature (°C)	0.05	0.951	2.71	0.106	0.17	0.678	0.83	0.443	0.15	0.864	0.66	0.422
Electrical conductivity (mS m^−1^)	0.02	0.980	9.30	*0.004*	0.01	0.958	2.22	0.119	0.09	0.915	0.01	0.957
Wheat parameter												
Germination rate (%)	0.51	0.601	0.01	0.998	0.51	0.477	1.25	0.295	0.51	0.601	0.03	0.859
Height (cm)	2.11	0.133	93.77	<*0.001*	0.06	0.799	0.47	0.627	0.06	0.945	0.85	0.362
Biomass (g)	0.87	0.424	3.84	0.056	0.14	0.705	0.21	0.815	0.69	0.506	0.53	0.472

No data available

Significant effects in italics; model degrees of freedom: seed dressing df = 2, earthworms df = 1, collembola df = 1

**Fig. 3:**
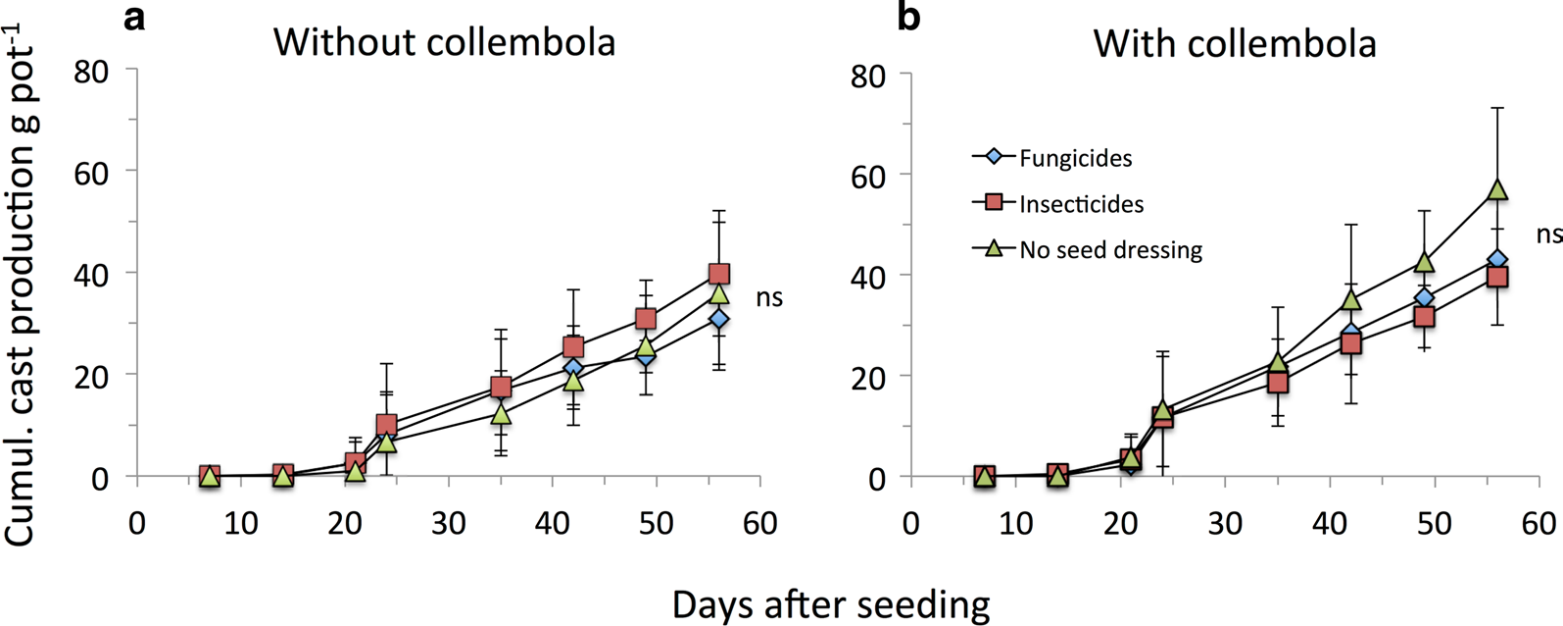
Earthworm surface activity in response to pesticide seed dressings in microcosms without (**a**) and with collembola (**b**). Mean ± SD, n = 5; ns denote no significant difference between seed dressings

Earthworms significantly reduced the surface activity of cumulative collembola activity (Fig. 1; Table 1), reduced soil basal respiration regardless of seed dressing (Fig. 2a) and reduced microbial biomass only when seed dressing was used (Fig. 2b). Additionally, earthworms increased soil water content and soil electrical conductivity (Tables 1, 2). Collembola significantly increased earthworm surface casting activity (Fig. 3; Table 1) and increased soil basal respiration (Fig. 2a; Table 1). Interactions between seed dressing and earthworms or between earthworm and collembola affected soil qCO_2_ (Table 1).

**Table 2:** Soil water content, soil electric conductivity and soil temperature in response to seed dressings, earthworms and collembola

Seed dressing	Soil water content (%)				Soil electrical conductivity (mS m^−1^)				Soil temperature (°C)			
	−EW		+EW		−EW		+EW		−EW		+EW	
	−C	+C	−C	+C	−C	+C	−C	+C	−C	+C	−C	+C
No seed dressing	8.07 ± 1.68	7.73 ± 0.93	8.86 ± 2.36	11.11 ± 2.32	0.62 ± 0.04	0.61 ± 0.05	0.97 ± 0.66	1.02 ± 0.22	16.43 ± 0.81	16.62 ± 0.61	16.79 ± 0.44	16.46 ± 0.56
Neonics	8.46 ± 1.04	9.11 ± 1.06	11.37 ± 2.25	10.65 ± 0.49	0.74 ± 0.10	0.71 ± 0.06	0.88 ± 0.18	0.84 ± 0.10	16.43 ± 0.52	16.67 ± 0.30	16.72 ± 0.60	16.53 ± 0.44
Fungicide	8.33 ± 098	9.49 ± 0.59	10.01 ± 1.41	9.91 ± 1.49	0.74 ± 0.16	0.82 ± 0.34	0.82 ± 0.12	0.83 ± 0.12	16.44 ± 0.42	16.16 ± 0.60	16.76 ± 0.37	16.76 ± 0.40

ANOVA results	Soil water content			Soil electrical conductivity			Soil temperature		
Factors	df	F	P	df	F	P	df	F	P
Seed dressing	2	1.976	0.150	2	0.022	0.978	2	0.070	0.933
Earthworms	1	20.826	<*0.001*	1	8.424	*0.006*	1	2.521	0.119
Collembola	1	1.518	0.224	1	0.026	0.872	1	0.193	0.662
SD × EW	2	0.904	0.412	2	2.490	0.094	2	0.846	0.436
SD × Coll	2	0.535	0.589	2	0.132	0.876	2	0.125	0.883
EW × Coll	1	0.000	0.983	1	0.001	0.980	1	0.681	0.413

ANOVA results for main factors and their interactions given below. Significant results in italics

**Fig. 4:**
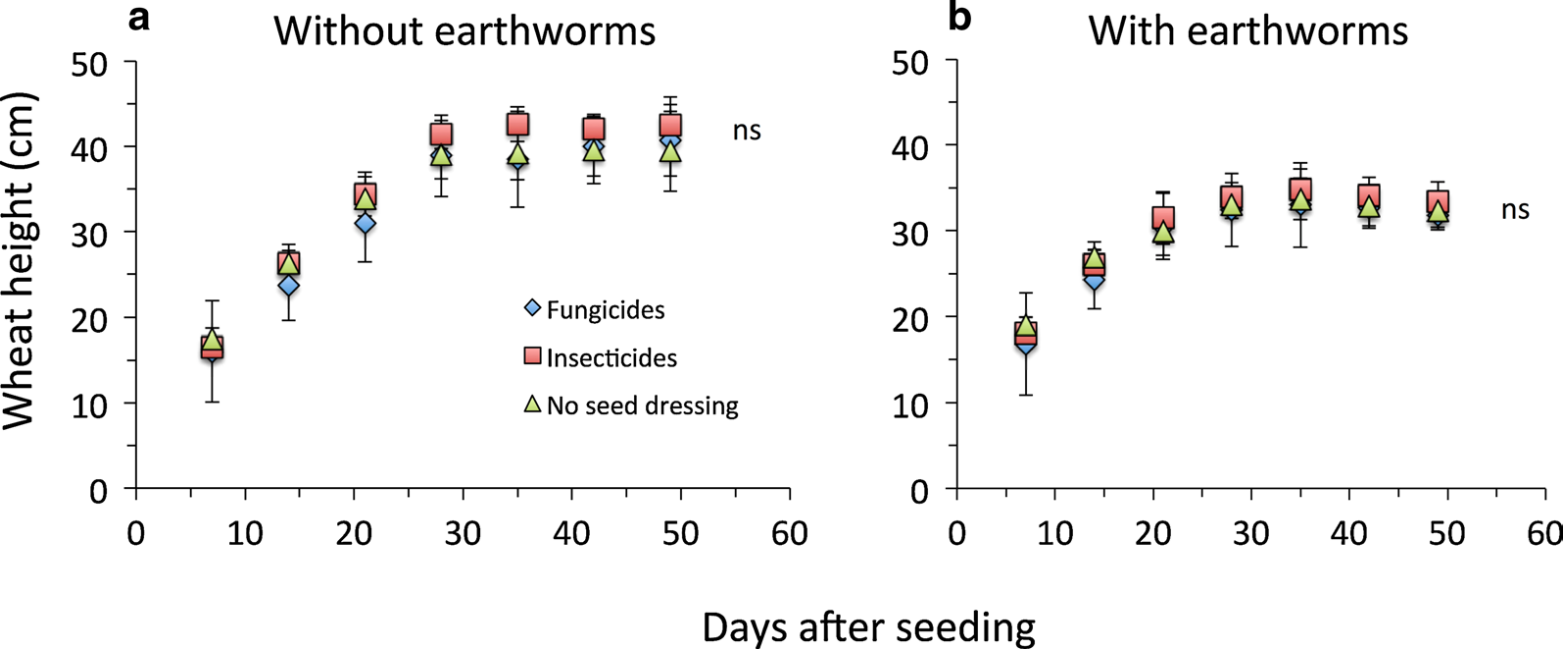
Wheat height growth in response to pesticide seed dressings in microcosms without (**a**) or with earthworms (**b**). Mean ± SD, n = 5; *ns* denote no significant difference between seed dressings

The average germination rate of wheat seeds among treatments was 91.9 ± 9.3 %, however this was not affected by any treatment factor (Table 1). Wheat growth was significantly and wheat biomass marginally significantly reduced by earthworms, however wheat growth was not affected by seed dressing or collembola (Fig. 4; Table 1). The mean final height of wheat was 33.8 ± 2.3 cm at 0.83 ± 0.30 g biomass when *L. terrestris* was present and 43.2 ± 3.5 cm at 0.69 ± 0.14 g without *L. terrestris* (Fig. 4).

## Discussion

This is among the first studies investigating realistic dosages of pesticide seed dressings on the activity of a variety of soil organisms and their consequence for ecosystem functioning exemplified by plant litter decomposition and crop growth. We found that fungicide seed dressings increased the activity of collembola and both insecticide and fungicides seed dressings increased the abundance of flagellate protozoa but decreased litter decomposition. Earthworm activity was not affected by seed dressings, however earthworms altered the response of collembola and soil microorganisms to seed dressings (i.e. seed dressing x earthworm interactive effects).

Soil fauna actively contributes to litter breakdown by grinding plant residues and thus increasing the surface area where bacteria and fungi actively mineralize carbon and nutrients [52, 53]. In our experiment litter decomposition rate was reduced by seed dressings, regardless whether insecticides or fungicides were used. As fungicides were also combined with the neonicotinoid insecticide seed dressings in the commercial seed material we used in the current experiment this indicates that neonicotinoids present in seed dressings had no additional effect on litter decomposition. The mesh size of the teabags we used (0.25 mm) also prevented the direct contribution of meso and macrofauna to litter breakdown [44], making the insecticide perhaps less relevant. Overall soil microbial biomass and activity was not affected by seed dressings suggesting potential shifts in soil fungal community composition rather than overall decrease in microbial (fungal) biomass and an increased nutrient input by decomposing fungi [54]. Our finding of reduced litter decomposition rates due to seed dressings could also be explained by increased protozoa abundance as protozoan grazing has been shown to affect the bacterial community structure in soil microcosms [55]. This assumption is further underpinned by strong increase in abundance of flagellate protists. Although flagellates may quickly respond to environmental changes [56, 57] the strong increase in the abundance of flagellate protist is surprising and reveals an important impact of seed dressings on basic soil food web functioning. Especially mycophageous flaggelates may have increased resource availability or reduced competition for resources that led to a twofold increase of flagellate cells. To the best of our knowledge, the present study is among the first ones reporting effects of pesticide seed dressings on protozoa. With abundances of several 100,000 individuals g^−1^ soil protozoa are at the base of the heterotrophic eukaryotic food web and an essential component in soil ecosystems because they consume a significant portion of the bacterial productivity, enhancing nutrient cycles and energy flows to the benefit of microorganisms, plants and animals [58–61]. Protozoa are also important grazers of rhizobacteria and can even influence aboveground herbivores [62].

In contrast to our hypothesis that collembola are strongly sensitive to insecticide seed dressing due to their close phylogenetic relationship to insects, seed dressings that only contained fungicides more strongly impacted collembola with an 250 % increase in surface activity and a 40 % increase in their reproduction rate. A higher surface activity of collembola might also be the consequence of an avoidance of soil areas contaminated with insecticide treated seeds. To what extent this can be interpreted as a reaction to chemical stressors needs to be investigated in specific behavioural experiments. Indeed, others also found an increased surface activity of collembola after application of seeds dressed with the neonicotinoid insecticide imidacloprid in the field [63]. Similary to flagellates, fungizide seed dressings may have increased resource availability for collembola, e.g. by increasing abundance of fast growing fungi that contain less toxins [64]. When fungicides and insecticides were sprayed, collembola were especially vulnerable [65] and have long been used as indicator species to asses non-target effects of agrochemicals [66].

Although, micro and mesofauna was affected by seed dressings, we found no clear effect on the casting activity of earthworms. This is a remarkable finding as earthworms are also known to feed on plant seeds [67–69]. In contrast, lethal and sublethal effects of neonicotinoid insecticides on earthworms have been documented by several studies [20, 26, 27]. However, these studies either considered sprayed insecticides and/or only tested the active ingredients while in the current study the complete formulations used by farmers, i.e. active ingredients including all (often non-declared adjuvants), were tested.

Earthworms altered effects of seed dressing on collembolan surface activity. We assume that the physical disruption by earthworm activity provided more hiding space and shelter for collembola hence mediating pesticide effects on collembola and also resulting in less collembola caught in pitfall traps. The effects of earthworms on the abiotic and biotic properties of their environment [70] may also have deluded local impact of seed dressings, however this also reflects organismic interrelationships present in agroecosystems. Additionally, earthworm activity also reduced protozoan abundance in presence of seed dressings suggesting shifts in organismic interactions due to seed dressings. Earthworms and collembola also affected soil basal respiration suggesting that negative effects of seed dressing on decomposition rate might have been counterbalanced by microbial activity. Remarkably in the current study earthworms decreased wheat growth, which is in line with [71] and might be due to feeding activities on roots [35, 72]. Soil water content was significantly increased in the microcosms containing earthworms which is probably a result of the decreased plant growth due to earthworm activity [22, 23] and thus a decreased transpiration of the winter wheat plants leading to higher soil moisture.

## Conclusions

Our findings suggest that pesticide seed dressing of wheat not only influence abundances and activities of soil micro- and mesofauna but might also alter nutrient cycling (via litter decomposition) with potential consequences for the functioning of agroecosystems. Soil macrofauna (earthworms) activity appeared to be less affected by seed dressings. This study is a first attempt to investigate potential non-target effects of seed dressings under more realistic circumstances including organismic interactions rather than only testing specific isolated active ingredients in laboratory settings. The tested effects of seed dressings on soil biota indicate that complex interspecific interactions such as resource- and interference competition may influence the assessment of non-target effects of pesticides. The reported effects may seem subtle, however it has to be noted that they were observed after a one-time application of only 18 seeds per experimental unit. However, under real farming conditions pesticide dressed seeds are sown on the same field at least twice a year with accumulating pesticide levels in soils [2] and potentially more pronounced non-target effects and feed backs on the composition of soil biotic communities and agroecosystem functioning [73]. Clearly, long-term field investigations are needed to further clarify potential effects of agrochemicals used for seed dressings on non-target soil organisms.
